# Development of a patient-led clinic visit framework: a case study navigating a patient’s journey for rheumatology outpatient clinic consultations in England and Wales

**DOI:** 10.1186/s41927-022-00318-3

**Published:** 2022-11-26

**Authors:** Sarah Oyebanjo, Paul Amlani-Hatcher, Ruth Williams, Roger Stevens, Tom Esterine, Kate Wilkins, Clare Jacklin, Jill Hamilton, Rosie Fairfax, Heidi Lempp

**Affiliations:** 1 British Society for Rheumatology, Bride House, 18-20 Bride Lane, London, EC4Y 8EE UK; 2 NRAS, NEIAA Patient Panel, Maidenhead, UK; 3 Department of Inflammation Biology, Centre for Rheumatic Diseases, Faculty of Life Sciences and Medicine, King’s College London, Cutcombe Road, 10, Cutcombe Rd, Weston Education Centre, London, SE5 9RJ UK; 4 NASS, NEIAA Patient Panel, London, UK; 5 National Rheumatoid Arthritis Society, Ground Floor, 4 Switchback Office Park, Gardner Road, Maidenhead, SL6 7RJ Berkshire UK; 6 National Axial Spondyloarthritis Society, 172 King Street, Hammersmith, London, W6 0QU UK; 7 Architects Registration Board, 8 Weymouth Street, London, W1W 5BU UK

**Keywords:** Axial spondyloarthritis, Early rheumatoid arthritis, Inflammatory arthritis, National audit, Patient and public involvement, Rheumatology

## Abstract

**Background:**

Involving patients and members of the public in healthcare planning is beneficial for many reasons including that the outcomes focus on topics relevant to service users. The National Early Inflammatory Arthritis Audit (NEIAA) aims to improve care quality for patients with inflammatory arthritis.

**Case study:**

This paper presents a case study detailing how the NEIAA Patient Panel worked with NEIAA governance groups, the National Rheumatoid Arthritis Society and the National Axial Spondyloarthritis Society to co-create an outpatient clinic visit framework for rheumatology professionals. A framework was co-created, divided into nine sections: pre-appointment preparation, waiting area (face-to-face appointments), face-to-face consultations, physical examination, establishing a forward plan, post consultation, annual holistic reviews, virtual appointments and key considerations. Providing insight into how the multi-disciplinary team can meet the diverse needs of patients with inflammatory arthritis, this framework now informs the teaching content about people who live with physical and mental disability for Year 3 and 4 undergraduate medical students at King’s College London.

**Conclusion:**

Patients play an important role in helping to address gaps in health service provision in England/Wales. The co-production of a clinic visit framework, informed by their own lived experience and their own expectations can lead to improved and relevant outcomes for the benefit of patients and raises awareness to medical students what matters to patients with physical disabilities when attending outpatient care.

**Supplementary Information:**

The online version contains supplementary material available at 10.1186/s41927-022-00318-3.

## Background

The benefits of involving patients and the public in research, service development and healthcare planning have been widely documented [1–6]. Some of these benefits include development of resources that are relevant to service users [1], enhancement of service delivery [4], patients feeling empowered [7], and change in organisational culture to further encourage patient participation in service re-design [6].

The National Early Inflammatory Arthritis Audit (NEIAA) is part of the National Clinical Audit and Patient Outcomes Programme (NCAPOP) commissioned by Healthcare Quality Improvement Partnership (HQIP). HQIP commissions, manages, supports, and promotes national and local programmes of quality improvement [8]. HQIP currently commissions 45 national audits [9]. Its aim is to promote quality improvement in patient outcomes, and, in particular, to increase the impact that clinical audit, outcome review programmes and registries have on healthcare quality. In 2018, the British Society for Rheumatology (BSR) was commissioned to continue to run the second phase of the NEIAA. The BSR is the UK’s leading specialist medical society for rheumatology and musculoskeletal healthcare professionals, supporting members to deliver the best care for their patients [10].

The purpose of the NEIAA is to improve the quality of care for people living with early rheumatoid arthritis (RA) by measuring care provided, against seven quality statements (QS), determined by the NICE quality standard 33 [11]. Early inflammatory arthritis (EIA) generally relates to patients with symptom duration less than three months [12]. In addition, the NEIAA assesses the time to diagnosis for patients with inflammatory disease of the spine (axial spondyloarthritis, axial SpA), a subtype of inflammatory arthritis (IA) with comparable treatment paradigms [13].

A large body of research [14–19] has demonstrated that evidence-based treatment, if instituted early, can prevent functional disability and improve mental health in patients with EIA.

In 2019, the first annual NEIAA report was published and one of the recommendations highlighted the need to promote EIA pathways across the rheumatology sector in England and Wales [20]. Findings highlighted inequalities in all domains of care, with about 27% of services without a dedicated EIA clinic [21, 22]. Research shows that the presence of EIA clinics increases the probability of patients starting conventional disease-modifying antirheumatic drugs (cDMARDS) in a timely manner by 12% and patients seen in an EIA clinic were started on a cDMARD on average eighteen days sooner compared to patients seen in a general rheumatology clinic [20].

In addition, the Project Working Group (PWG) of the NEIAA suggested to the Patient Panel (see below) to co-create a clinic visit framework for England and Wales, based on their own experiences of receiving care and treatment in outpatient clinic settings. The aim of the document was to help each newly diagnosed patient how best to prepare and what to expect from outpatient clinic visits in secondary care, as part of their adaptation living with a long-term condition. The literature from a case study of patient involvement in outpatient service design [1] and a systematic scoping review of healthservice needs of patients [23] highlighted patients can help to improve services when supported and respected as equals, and the organisation is ready to implement change. Other important aspects are improved communication between patients and clinicians, characteristics of ongoing care, e.g. consultation length and continuity; factors influencing care seeking, e.g. disease severity, family expectations and input from allied healthcare professionals and availability of complementary and alternative medicine.

## Public and patient involvement (PPI) in NEIAA

At the inception of this national audit, BSR collaborated with Kings College London to recruit some of the members of the patient panel with IA. The Patient Panel coordinator was approached by a PWG member to invite members from the King’s College London Expert Patient Group, and some of the group members agreed to join the Patient Panel as they anticipated that their contributions were important, especially in improving care for patients with early RA. As such, the Patient Panel members have been involved as public contributors prior to NEIAA.

The Chair of the Patient Panel, who is a patient diagnosed with Rheumatoid Arthrititis, was a volunteer with NRAS who approached him to see if he would be interested in the role. After discussing the position with the NEIAA clinical director, he agreed to take on the position. The panel’s Vice Chair, who is also a patient with Axial Spondyloarthritis, was a volunteer for NASS who approached him about the position. Their respective experiences of receiving care in the NHS have been instrumental in their appointment for the NEIAA.

The Patient Panel is diverse both in relation to socio-demographics (sex: 6 female/3 male, age: 37–72 and to some extent ethnicity 1 Black/8 White,) and their disease experiences (1 Ankylosing Spondylitis/8 Rheumatoid Arthritis). All live with symptoms of pain, stiffness, fatigue and at times mental distress, and receive a combinations of DMARDs and biologics. All had a diagnosis of their musculoskeletal conditions for more than 5 years, i.e. all have an established rheumatological condition. One has hips, both wrists, and both thumbs replaced/fused. Members live and receive treatment from different geographical areas in England, covering Portsmouth, Bristol and London. The majority of Patient Panel members are not in salaried employment due to their rheumatological conditions.

Support was offered to Panel members in terms of accessibility of meetings and training. Meetings were always very well organised in advance, with an agenda and minutes, and members had the opportunity to attend virtually, also prior to the COVID-19 pandemic. Travel was re-imbursed during face-to-face meetings and refreshments were offered. Remuneration is not currently possible, but the NEIAA team have reviewed this situation, and compensation will be made available in the new contract.

In addition, the NEIAA Governance Groups have representation from two patient organisations; the National Rheumatoid Arthritis Society (NRAS) and the National Axial Spondyloarthritis Society (NASS).The Patient Panel is an independent group that meets regularly, initially face-to-face and since the outbreak of COVID-19, online with an agenda either set by the group or through requests from the NEIAA Project Working Group (PWG) or Senior Governance Group (SGG) and feeds back decisions to the different governance meetings via their chair, vice-chair, and patient panel coordinator.

## Contributions of the Patient Panel to the NEIAA

Patient reported outcome measures (PROMs) are employed in healthcare to assess the quality of care provided from the patient perspective [24] e.g. The Health Assessment Questionnaire (HAQ) Disability Index [25] and Bath Ankylosing Spondylitis Diseases Activity Index (BASDAI) [26] and Visual Analog Scale (VAS) [27]. The outcomes assessed are usually of importance to patients and thus measuring patient reported outcomes (PROs) are linked with higher patient satisfaction [28]. A major success of NEIAA, to date, has been the capture of PRO quality measures (e.g. disease impact, work, mental health). A separate patient portal [29] was built to enable direct data entry from patients. The Patient Panel was involved in piloting the platform to ensure the content and functionality was relevant to patients and fit for purpose. The Patient Panel also supported the development of the PRO data download tool which reinforces the value of submitting PRO data to patients as well as empowering patients to share their own care decisions with clinical staff. Furthermore, the Patient Panel ensures that findings from the audit data collected are available in an accessible format for patients and members of the public and thus contribute to the writing up of the lay Annual Patient and Public Report [30]. Five of the Patient Panel members also co-authored this article. The Group’s contributions are shown in Table 1.

**Table 1:** Contributions of the Patient Panel to different workstreams

Date	Activity
May 2018	Patient portal feedback and development The group reviewed the content included on the platform and shared their feedback with the audit team to allow them to make the content and navigation more patient friendly. They also suggested functionality that would be useful for patients
April 2019	BSR Annual Conference The Patient Panel chair presented about the importance of including the Patient Reported Outcomes (PRO) in the audit
October 2019	Writing the first annual patient and public report The group co-produced the report with the Project Manager identifying content relevant to patients and ensuring that the information was presented in a format suitable for patients and members of the public
October 2019	Training for participation in BSR initiated rheumatology service review Group members involved in the Quality Review Scheme received training on how to effectively review rheumatology services. They were given opportunities to feedback on the process
April 2020	BSR Annual Conference The Patient Panel Chair presented findings from the first annual report focusing specifically on the PRO to the rheumatology multi-disciplinary team
June 2020	Disease activity score functionality on the webtool The group reviewed the new functionality added to the NEIAA patient portal allowing patients to examine and report on swollen and tender joints and provided feedback to the NEIAA team that led to necessary changes to be made
August 2020	NEIAA supplementary report webinar speaker [31] The Patient Panel Chair presented findings from the supplementary report to the rheumatology multi-disciplinary team
October 2020	Quality improvement plan The group reviewed the QI plan ensuring that the content was focused on improvement objectives that would ultimately improve the quality of care provided to patients in England and Wales, e..g. preparing and sharing resources with the MDT
November 2020	HQIP Annual General Meeting presentation The Patient Panel Chair presented the involvement of the Patient Panel in the audit
January 2021	Writing the second annual patient and public report The group co-produced the report with the Project Manager identifying content relevant to patients and ensuring that the information was presented in a format suitable for patients and members of the public
February 2021	NEIAA second annual report webinar The Patient Panel Chair presented findings relating to patients from the second annual report to the rheumatology multi-disciplinary team
April 2021	Podcast discussing journal paper [32] In the recording Dr Marwan Bukhari talks to Paul Amlani-Hatcher (NEIAA Patient Panel Chair) and Dr James Galloway (King’s College, London) about findings from the NEIAA. After introducing the audit, Dr Galloway discusses predictors of disease activity, while Paul Amlani-Hatcher explains how the data may help to inform and empower people with inflammatory arthritis
April 2021	BSR annual conference The Patient Panel Chair presented findings from the second annual report focusing specifically on the patient reported outcomes to the rheumatology multi-disciplinary team The presentation was entitled “Clinician and pro data collection—why is this important” and was part of a wider session on NEIAA. The presentation was drafted by the Patient Panel Chair with input from the other panel members and other speakers in the session. The overriding message was to present the patient focus on what was important in the findings with a focus on mental health and employment impacts. There were several follow up questions from the audience seeking further clarity on the points made—the Patient Panel Chair responded to these questions “live” in the session
June 2021	Clinic visit framework The group led on the production of the framework described in detail in this paper, based on their lived experiences attending a range of different outpatient clinics with positive and negative aspects
January 2022	Reviewing and providing feedback for the short report on ethnicity Several Patient Panel members reviewed the content of the report and provided feedback on the data presentation and information included in the document. Also, one of the Patient Panel Members described his personal experience about his care at a rheumatology outpatient clinic when he was diagnosed with RA and its impact on his life living with a long-term condition

Members of the Patient Panel all have extensive experience (> 5–> 50 years) of attendance outpatient rheumatology services in the NHS across England and Wales. This ‘expertise’ was harnessed by the PWG and will be the focus of the case study. The aim of the task was the co-creation and development of a national Clinic Visit Framework [33] led and written by members of the Patient Panel, within a consortium of the NEIAA multi-disciplinary PWG, SGG, head of quality improvement and two national patient organisations (NRAS & NASS).The group produced the final framework over 12 months.

## Case study

The following stages outline the development of the framework:

**Preliminary stage**: Brainstorming

Prior to the Patient Panel meeting (March 2020), research was conducted by the NEIAA Project Manager to identify gaps in resources available to support rheumatology multi-disciplinary teams (MDTs) when delivering outpatient care. This process ensured that the group was aware of what information was already available in the public domain and prevented duplication of efforts. With the available information in mind, the first meeting of the group took place to clarify the scope, identify the structure, sections and content of the framework. The Patient Panel members were responsible for leading this process and decided on the structure and relevant content to be included in the framework, based on their lived experiences. Patients were not contributing to a pre-determined activity, however the preliminary work acted as a foundation from which the framework could be shaped and completed in time, rather than starting with a blank sheet.

During this stage, the group was encouraged to share their ongoing experiences when attending outpatient care and any barriers encountered in open discussions guided by a mutually agreed structure.The group discussed several barriers experienced at different stages in their care pathway. The hurdles  raised were used to inform the different sections outlined in the framework. Difficulties mentioned prior to the appointment included a lack of clarity of the exact location of the appointment. Some of the patient panel members reported that they were confused when trying to locate where their appointment was going to take place. Another challenge was about the structure of the appointment. There was lack of information on what would be covered during the appointment. They said that it would be useful to know in advance other departments that they would be visiting on the day of the appointment. This can help them with time management and feeling more prepared during the appointment.

A few challenges were noted in the waiting area. These include uncomfortable seating area and poor signage of the section for rheumatology patients when it was a shared waiting area. Some patients had experienced poor communication during their appointments, e.g., no prior knowledge when their consultant or nurse was running late with their appointment that prolonged the stressful waiting in overcrowded waiting areas. Experiences noted during face-to-face appointments included the clinicians failing to make regular eye contact with the patient, spending too much time looking at the screen to seek results of investigations, e.g. blood test results, X-rays etc.; thus failing to build a rapport with the patient. Commonly, the clinician failed to check the patient’s level of understanding of the information provided thus leaving them feeling overwhelmed or confused.

**Stage 1:** Outline and structure of document

Following on from the first meeting, information was gathered to inform the framework [33] (June 2020). Based on the key themes collated, the structure was put into nine sections mirroring a patient pathway. These sections were pre-appointment preparation, waiting area, face-to-face appointment, face-to-face consultations, physical examination, establishing a forward plan, post-consultation, annual holistic reviews, virtual appointments and key considerations.The content was then shared with the group for comments and feedback (July 2020). The Patient Panel chair and coordinator discussed the initial plans for the framework at the SGG and PWG meetings (September and October 2020 respectively) to gather any initial feedback or comments from respective, mainly clinical involved healthcare professionals (see other meetings attended in Table 2). The overall response was positive, as the content was realistic and reflected well what patients expected when attending outpatient clinics.

**Table 2:** Showing meetings attended by the Patient Panel

Meeting date	Meeting title
3 April 2020 3 July 2020 12 October 2020 15 January 2021 19 April 2021	PWG meeting
14 May 2020 15 September 2020 17 November 2020 10 February 2021 11 May 2021	SGG meeting
19 March 2020 12 June 2020 26 February 2021	Patient panel meeting

Meetings attended by patient panel representatives between March 2020 and June 2021 (clinic framework publication)

**Stage 2:** Alignment of content to BSR Quality Standards

The document went through several iterations (October–December 2020) based on feedback received from the Patient Panel.Examples of feedback received from the Patient Panel included:

- ‘When preparing for an appointment, patients need to  be informed to wear comfortable and easy to remove clothing for examinations, as this consideration is often overlooked when planning the visit’, specifically when patients go on, or come to the clinic from their place of work. This was added into the framework under the heading ‘pre-appointment preparation, 1.6.
- Patients appreciate when clinicians introduce other staff or medical/allied health professional students present at the appointment, so the patients know who else is attending and their reasons, e.g., learning about rheumatology care, communication. The patient can then express a choice whether the presence of others is appropriate at the time or not. This was added into the framework under the heading Face to face consultations, 3.2.
- Patients welcome to be asked about their mental wellbeing and how the long-term musculoskeletal condition has affected them. It is also important to consider how their condition impacts on their home circumstances, relationships and salaried or voluntary work. This was added into the framework under the heading Face to face consultations, 3.9.

Following the final agreement on the structure and content of the framework, including an additional section about online consultation during the COVID-19 pandemic, the document was shared with the BSR Head of Quality Improvement (QI) who suggested mapping the content against the organisation’s Quality Review Standards (QRS) [34] for consistency. The Head of QI reviewed the framework and shared suggested changes with the group. (December 2020). Each inconsistency was discussed with the Patient Panel and each member had the opportunity to agree or disagree with the proposed changes. The group agreed with all the changes proposed. An example of a suggested change was the number of days for the consultation summary letter to be sent to the GP/patient. The Patient Panel had suggested that this needs to be completed within five days. However, the QRS measures state that consultation letters need to be sent within 10 working days. Another example was that the framework mentioned that patients prefer to have access to the relevant MDT members at the time of appointment. The corresponding QRS measure states that patients need to have access to a member of the MDT following initial medical assessment.

**Stage 3**: Obtaining additional information from National Patient Organisations

A meeting then took place (February 2021) to discuss the suggested amendments in detail and the group was given an opportunity to approve or decline the changes. The document was then shared with NRAS and NASS (March 2021) who reviewed the content and added further important links and relevant information for patients/carers/health professionals.

**Stage 4:** Approval of the framework by the NEIAA Governance Groups

The updated framework was shared with the PWG and SGG for further comments. (April 2021). The PWG and SGG raised several useful points for consideration including a disclaimer stating that the authors were aware of the challenges currently faced by rheumatology teams, as a result of the pandemic. The PWG Vice-Chair had been involved in producing a guidance document on virtual appointments [35] and suggested that the group review the content for additional insertions. The framework received positive opinions by both groups and was endorsed as a highly valuable clinical framework for rheumatology teams and patients/carers. One clinician in the PWG stated “this document is so valuable for me as a clinician, I had no idea that all the sections are so important to patients, a real eye opener”.

## Impact of the clinic framework

This framework provides further evidence how best MDTs can meet the needs of patients when attending rheumatology outpatient clinics in England and Wales. With findings from the NEIAA second annual report showing that when patients first presented with EIA, the burden of mental health was higher than at 12 months, this highlights the impact that the MDT could have on patient outcomes [28]. Access to the relevant services such as psychology therapies can help to improve an individual’s mental health and reduce the likelihood of absenteeism. The association between work loss and absenteeism with IA is well known [36, 37] and the link could have wider reaching impact on the individual’s physical and mental health. The psychological impact and risks of work loss / absenteeism of EIA are likely to be emphasised further by the current delays (diagnosis /referral) relating to the COVID-19 pandemic.

The document is currently utilised for the curriculum development of cultural competence in relation to physical and mental disability to teach Year 3/4 undergraduate medical students in King’s College London. As the content was written directly by patients the medical educators and patient educators who teach on the module decided that students at an early stage of their learning need to be aware what matters to patients with disability and enhance their clinical and communication skills, professional attitude and behaviour when providing medical care and treatment in the future.

To ensure maximum reach of the clinical framework various media have been targeted, e.g. the BSR monthly newsletter, the NEIAA newsletter, NRAS magazine and newsletter, NASS magazine and newsletter, social media and BSR, NRAS and NASS websites. Collectively, channels have a reach of over 180,000 people. Since publication in June 2021, the document has been opened 568 times on the BSR and NRAS websites for example.

Two of the Patient Panel members attend the PWG and SGG meetings to ensure that the patient voice is considered in every workstream of the NEIAA. For example, following publication of the annual report, the chair of the Patient Panel presented at webinars and the BSR annual conferences in April 2020/2021 about the importance of improving service provision for patients with their direct input.

On an annual basis, HQIP invites applications for The Richard Driscoll Award to acknowledge public and patient involvement in national clinical audits. In 2021, HQIP requested submissions showcasing excellent patient and public involvement. The NEIAA received commendations in 2019 and 2021, having won the award in 2020. Feedback from the review panel included “that there were several examples of co-production with evidence of a robust patient panel and great use of the framework for teaching purposes” (Additional file 1).

As well as improving services and health outcomes, this framework also presents an opportunity to empower patients [7] (see Table 3). The Co-patient Panel Chair noted in the Richard Driscoll Award submission 2021 that “The NEIAA has offered me an opportunity, as somebody recently diagnosed with RA, to add my voice and that of other people I am in contact with through NRAS to this amazing audit. As a patient I have also been able to access the data for my Health Provider and gauge their performance, in responding to IA, up against other health providers as well as the quality standards set out by NICE. The audit has given me a level of knowledge about how IA is being managed nationally and locally that I am able to broadcast through other channels of my volunteering activity in this area of health provision” (Additional file 2).

**Table 3:** Highlighting reasons by Patient Panel members for becoming involved in the audit [38]

Patient Panel Member 1	'I joined the BSR Patient Panel as I felt that I could offer a particularly useful input given that my diagnosis for RA was relatively recent, in 2015 I feel that I benefited from the early referral as my RA is now in remission and I have practically no visible joint damage. I even remember my rheumatologist saying that I was part of the pilot for the NEIAA new reporting'
Patient Panel Member 2	‘As soon as I heard about the NEIAA work I was keen to be involved. The NEIAA will help to provide a stimulus to help improve diagnosis and treatment for all types of IA and if I could help in a small way to assist the work of the project, I am happy to do so I owe my mobility to the superb work of the NHS rheumatology team and physiotherapists at Queen Alexandra Hospital, Portsmouth, and this is a way of giving something back'
Patient Panel Member 3	‘After being diagnosed I felt I needed to find out everything there was to know about a condition which initially impacted me greatly. Consequently, I wanted to become involved with all things relating to my condition, something I felt would give me back my self-esteem, given the initial impact With this lived experience, therefore, I now have the opportunity and, indeed, want to contribute towards anything that makes patients' lives a little less challenging while having to deal with their condition'
Patient Panel Member 4	‘My involvement with NEIAA is because I think patient input is vital for all aspects of research and patient care. I have had RA for many years and there have been huge advances in medication for most auto immune conditions, but patient involvement in research and teaching lags behind Being part of a like-minded group is energising and makes use of my brain which still functions, despite physical restrictions!'
Patient Panel Member 5	'I became involved with the audit, because I have lived with RA for most of my life, having been diagnosed in early childhood. I have received many different therapies over the years and seen a huge improvement in treatment effectiveness I realise how very important it is for all patients to receive effective treatment early on in their disease, to prevent joint damage and disability. However, I know that sadly this does not always happen. I believe the audit will help highlight why this might be and enable us to improve care pathways, both locally and nationally in order to keep people healthy, active and in work whenever possible'

## Discussion

This clinic framework has given patients the opportunity to contribute systematically to an agreed work package that will benefit them and others now and in the future. Patients are the experts living with their conditions and listening to their voices, ideas and concerns, is key in any service development and improvement [1]. From the experience of the NEIAA Patient Panel, it is clear that to increase patient involvement in clinical audit, it is important for patients to feel that they can contribute openly to discussions without feeling as though they are simply complaining about their lived experience. It is also important that Patient Panel members’ skills are recognised—whilst they are patients, they are also people with a rich diversity of life and employment experience.

Going forward, the group will continue to contribute to different workstreams in the NEIAA to improve the patient pathway for early RA and axial SpA service provision. For example, Patient Panel members have recently initiated and contributed to the NEIAA short report on ethnicity, focusing on the association between ethnicity and health outcomes [39]. Within the report one of the patient panel members shared their experience from receiving a diagnosis in August 2011 and the subsequent treatments received following an unexpected diagnosis that was life changing.

## Future plans

The Patient Panel will continue to contribute to the audit in the following ways:

(i) Attend PWG and SGG meetings to provide insight on the patient perspective
(ii) Attend Patient Panel meetings to discuss items relevant to the group and work on projects relevant to the NEIAA, e.g. BSR Service review
(iii) Review and provide feedback on current NEIAA workstreams
(iv) Contribute to the development of the patient and public annual report
(v) Review and update the clinic framework

## Limitations of the framework

With a small sized patient panel, it is likely that some key aspects within the framework would have been missed, e.g. pregnancy and ante-natal care, needs of young people up to 25 years. The involvement of two national patient charities helped to broaden out the collective feedback from patients that made the content of the framework more relevant and real.

As the document was produced and published during the Coronavirus pandemic, it was important for the group to be sensitive to the pressures experienced by rheumatology teams in England/Wales. As mentioned above, a disclaimer was included in the document acknowledging with the current pressures on services due to redeployment of staff and sickness levels, it is challenging to achieve some of the quality standards.

## Conclusion

Patient centred care is more likely to be be associated with improved health outcomes. Patients can help to expand and enhance service provision by contributing to the development of resources for rheumatology professionals. The literature highlights that patients are willing to help with outpatient service design [1] and the Patient Clinic Framework document includes many dimensions patients perceive as important when attending rheumatology outpatient clinic services [23], e.g. doctor-patient communication, input from Allied Healthcare Professionals, evidence-based treatment and others. The Patient Clinic Framework is comprehensive in its content and can assist clinical staff in their ongoing care and support with patients living with musckucloskeletal conditions and respond to their specific bio-psychosocial needs when attending outpatient care.

As this case study focuses on England/Wales, there may be some limitations with its utility in other geographical areas. Effective engagement means involvement from the inception of a new initiative and throughout the duration of the national audit to capture what matters to service users. Further work needs to be carried out to monitor the impact of the framework on rheumatology teams and whether the information in the document is being implemented successfully over time.

### Supplementary information

NEIAA: clinic visit framework (https://rheumatology.org.uk/Portals/0/Documents/Practice_Quality/Audit/NEIA/Patient-clinic-visit-framework.pdf?ver=2021-06-14-215653-737).

## Supplementary Information

**Additional file 1.** Plain Language Summary.

**Additional file 2.** GRIPP-2 checklist.

## Data Availability

Data sharing is not applicable to this article as no datasets were generated or analysed during the current study.

## Abbreviations

BASDAI: Bath Ankylosing Spondylitis Diseases Activity Index
BSR: British Society for Rheumatology
cDMARDS: Conventional Disease-Modifying Antirheumatic Drugs
EIA: Early inflammatory arthritis
HAQ: The Health Assessment Questionnaire
HQIP: Healthcare Quality Improvement Partnership
IA: Inflammatory Arthritis
MDT: Multi-Disciplinary Team
NASS: National Axial Spondyloarthritis Society
NCAPOP: National Clinical Audit and Patient Outcomes Programme
NEIAA: The National Early Inflammatory Arthritis Audit
NICE: National Institute for Health and Care Excellence
NRAS: National Rheumatoid Arthritis Society
PROMs: Patient Reported Outcome Measures
PROs: Patient Reported Outcomes
PWG: Project Working Group
QI: Quality Improvement
QRS: Quality Review Standards
QS: Quality Statements
RA: Rheumatoid Arthritis
SGG: Senior Governance Group
VAS: Visual Analog Scale
