# Supplementary material for: Development of a patient-led clinic visit framework: a case study navigating a patient’s journey for rheumatology outpatient clinic consultations in England and Wales
Source: BMC Rheumatol. 2022 Nov 26;6:89. doi: 10.1186/s41927-022-00318-3 (PMC9700913; doi:10.1186/s41927-022-00318-3)
Supplement: Supplementary file 1 — Additional file 1. Plain Language Summary. [file 41927_2022_318_MOESM1_ESM.docx]

**Plain language summary**

Involving patients and members of the public in healthcare planning is beneficial for many reasons including that the outcomes focus on topics relevant to service users. The National Early Inflammatory Arthritis Audit (NEIAA) aims to improve care quality for patients with inflammatory arthritis (IA).

The NEIAA Patient Panel consisting of nine individuals from diverse backgrounds diagnosed with inflammatory arthritis. One coordinator provides support and advice to the group for the delivery of the NEIAA. This paper presents a case study detailing how the NEIAA Patient Panel worked with NEIAA governance groups, the National Rheumatoid Arthritis Society and the National Axial Spondyloarthritis Society to co-create an outpatient clinic visit framework for rheumatology professionals. Based on the themes raised by the Patient Panel, governance groups and the two national patient organisations, a framework divided into nine sections was created: pre-appointment preparation, waiting area (face-to-face appointments), face-to-face consultations, physical examination, establishing a forward plan, post consultation, annual holistic reviews, virtual appointments and key considerations.

As well as providing further insight into how the multi-disciplinary team can meet the diverse needs of patients with inflammatory arthritis, this framework informs the teaching content about people who live with physical and mental disability for Year 3 and 4 undergraduate medical students at King’s College London.

Patients play an important role in helping to address gaps in health service provision in England/Wales. It is vital that patients’ lived experiences are given the opportunity to contribute to the development of resources for professionals. Co-production will lead to improved and relevant outcomes.
