# Supplementary material for: Development of a patient-led clinic visit framework: a case study navigating a patient’s journey for rheumatology outpatient clinic consultations in England and Wales
Source: BMC Rheumatol. 2022 Nov 26;6:89. doi: 10.1186/s41927-022-00318-3 (PMC9700913; doi:10.1186/s41927-022-00318-3)
Supplement: Supplementary file 2 — Additional file 2. GRIPP-2 checklist. [file 41927_2022_318_MOESM2_ESM.docx]

**GRIPP-2 checklist for reporting PPI**

| **Section and topic** | **Item** | **Reported on page no** |
| --- | --- | --- |
| 1. Aim | Report the aim of PPI in the study | 3 |
| 1. Methods | Provide a clear description of the methods used for PPI | 4-6 |
| 1. Study results | Outcomes- Report the results of PPI in the study, including both positive and negative outcomes | 7 |
| 1. Discussion and conclusions | Outcomes- Comment on the extent to which PPI influenced the study overall. Describe positive and negative effects | 8-9 |
| 1. Reflections/ critical perspective | Comment critically on the study, reflecting on the things that went well and those that did not, so others can learn from this experience | 10/11 |
